# Open Source, Open Standards, and Health Care Information Systems

**DOI:** 10.2196/jmir.1521

**Published:** 2011-02-17

**Authors:** Carl J Reynolds, Jeremy C Wyatt

**Affiliations:** ^2^Institute for Digital Health careInternational Digital LaboratoryWarwick UniversityCoventryUnited Kingdom; ^1^Centre for Health Informatics and Multiprofessional EducationUCL Medical SchoolLondonUnited Kingdom

**Keywords:** Health Care Information Systems

## Abstract

Recognition of the improvements in patient safety, quality of patient care, and efficiency that health care information systems have the potential to bring has led to significant investment. Globally the sale of health care information systems now represents a multibillion dollar industry. As policy makers, health care professionals, and patients, we have a responsibility to maximize the return on this investment. To this end we analyze alternative licensing and software development models, as well as the role of standards. We describe how licensing affects development. We argue for the superiority of open source licensing to promote safer, more effective health care information systems. We claim that open source licensing in health care information systems is essential to rational procurement strategy.

## Introduction

Doctors, patients, and policy makers are increasingly aware of the significant improvements in patient safety, quality of patient care, and efficiency that health care information systems (HIS) have the potential to bring [1-3]. This has led to significant investment in HIS. Investment has also been motivated by a desire to capitalize on the global market for HIS, estimated to be worth US $53.8 billion by 2014 [4], by developing HIS for export. In the United Kingdom, contracts were negotiated in 2004 for a National Health Service (NHS) National Programme for Information Technology (NPfIT) with a budget of £12.4 billion over 10 years. This makes it an information technology (IT) project unprecedented in terms of cost and scale [5]. Furthermore, the current US administration has recently displayed the political will for wider adoption of HIS by committing US $19 billion to develop and encourage the implementation of HIS as part of the American Recovery and Reinvestment Act of 2009 [6,7].

However, difficulties have been experienced in the United Kingdom delivering the NPfIT on time and within budget [8]. Additionally, concern has been expressed that a lack of clinical engagement threatens the success of the project [5,9-12]. While some progress has been made with networks, hardware, and software, many promised benefits such as single-point data entry (“With IT, information can be captured once and used many times” – Downing Street 2002 NHS IT Briefing [13]) are still eagerly awaited by practicing UK clinicians [14]. In the United States, excepting the Veterans Administration (VA) hospitals’ HIS, uptake of HIS has been poor [7]. While it is too early to assess the results of the fiscal stimulus, concern has been expressed that the procurement process, standards, and certification will be biased in favor of software vendors who operate closed development models and sell their software with proprietary licenses. Furthermore, this may be to the detriment of rapid widespread adoption, and meaningful usage, of effective HIS [15].

We believe that open source software (OSS) licensed HIS provide a key opportunity for the promotion of effective systems by enhancing clinical engagement in software development, fostering innovation, improving system usability, and reducing costs, and should therefore be central to a rational HIS procurement strategy.

## Background

### Approaches to Software Development and Licensing: Proprietary Software and Open Source Software

In terms of software development and licensing, there are broadly two kinds of software: (1) proprietary software, such as Microsoft Internet Explorer Web browser, and (2) OSS, such as Mozilla Firefox Web browser.

**Figure 1:**
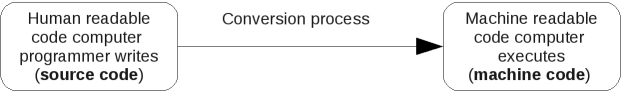
The programmer writes source code, which is converted to the machine code that the computer runs

The major difference between the two is the availability of the source code. This is the code computer programmers write, which is turned into the machine code computers execute (Figure 1).

OSS is software where the end user can access and modify this source code, because of their rights under the licensing arrangement, to make new machine code and redistribute it. With proprietary software the source code is secret and the end user can access and execute only the machine code.

In fact, the reality of OSS is more complex. We use OSS in this paper to refer to both Free/Libre, as in the sense of the French “libre”, software and open source software. This is also sometimes referred to as free and open source software. Whichever term is used, OSS refers to a large number of different software licenses that have certain requirements for source code openness in common [16]. The Free Software Foundation and the Open Source Initiative act as arbiters of these licenses.

Free software and OSS movements disagree on aspects of commercialism and licensing but agree on many fundamental principles such as the availability of the source code and the ability to modify and distribute it freely. Specifically, most free software licenses are less permissive. They forbid both contamination of source code with proprietary code, and later closure of source code previously released under an open license [17].

It is crucially important to realize that the quality of the software and source code is not inherently affected by the nature of the license. The application of a license to a piece of source code does not affect the code per se, but the type of license does affect the development of source code and has long-term implications for the purchaser.

### Open Standards Facilitate Competition Between Open Source Software and Proprietary Software

Having defined open *source* it is expedient to examine open *standards,* since it is often suggested that they, and not open source, should be required by a purchaser in order to promote competition between proprietary software and OSS.

Usage of the term *open standard* varies considerably. There is agreement upon what constitutes a standard, but disagreement on what is required for a standard to be considered open.

Standards may be classified according to their openness. Cerri and Fuggetta [18] give a useful system of classification, which we have adopted here.

1. Closed: the standard is owned by a company and is kept secret (eg, the Skype communication protocol).
2. Disclosed: the standard is owned by a company but is made available to other companies and users (eg, Adobe PDF format).
3. Concerted: there is a consultation on a new standard, but admission to the consultation process and management of the process is controlled by a company (eg, Sun Microsystems Java programming language).
4. Open concerted: there is an open participation in the process through which the standard is defined and managed (eg, World Wide Web Consortium [W3C] HTML).
5. Open de jure: the standards are owned and managed by official international or national standardization bodies (eg, the Digital Imaging and Communications in Medicine [DICOM] standard).

An open standard is developed through methods 4 or 5 and must fulfill all of the following requirements [18]:

1. The standard specification document must be publicly available, either free of charge or at a nominal fee.
2. The standard must be owned and managed by an official standardization body or by an open group or consortium. It must not be owned or controlled by a single party, and no single party must have special rights to it.
3. The standard must be defined and managed according to an open process. Every interested party must be able to join the standardization process, which must be based on an open decision-making procedure (eg, consensus).
4. The standard must be free to implement for all interested parties, without any royalty fee. Any patented technologies included in the standard must be licensed with royalty-free nondiscriminatory terms.
5. It must be possible to extend and reuse the standard in other open standards.

Evolution of the computer industry has been driven by the emergence of standardized platforms that allow, and even encourage, modular substitution of complementary components such as software and hardware. Briefly, this evolution charts the shifts in business strategies of the Big Four: Apple Computer, IBM, Sun, and Microsoft. With time, vertically integrated proprietary platforms such as early IBM mainframes gave way to horizontally specialized strategies of personal computers and servers. More recently, the emergence of OSS has necessitated further refinement of business strategy. Three of the Big Four have developed hybrid OSS and proprietary platforms and now emphasize selling services and support rather than software alone [19].

Standards matter to businesses, who are keenly interested in establishing dominant standards where possible, ensuring that their products interoperate with the dominant standard where not, and in any case influencing and using standards for their own benefit. This is why compatibility issues are frequently encountered when one tries, for example, to open a Microsoft Word 2007 document on a computer not using a Microsoft operating system, or even a computer not using the same version of Microsoft Word.

The aim of open standards is to have competing implementations of the same standard, rather than competing platforms, in order to benefit consumers. The rationale is that open standards lower entry barriers and encourage competing implementations of the same standard, which in turn tends to foster innovation and lower costs to the consumer. The consumer is empowered to change products without losing data or facing significant conversion costs, thereby preventing lock-in. Further, together with antitrust laws, open standards help to protect consumers from monopolies [18,19].

### Open Standards Need Open Source Software Implementations

Proper development and maintenance of an open standard requires a balance between not allowing extension, which may prevent evolution of the standard and stifle innovation, and allowing proprietary extensions, which can lead to the subversion of a standard [19].

An open standard can also be subverted where adoption of proprietary standard is sufficiently widespread for it to become a de facto rival standard. For example, Internet Explorer has introduced an array of proprietary extensions to many of the standards, such as HTML (maintained by the main international standards organization for the World Wide Web, the W3C). Consequently, webpages that make use of these proprietary extensions appear broken even in standards-compliant Web browsers, introducing the need for a “quirks mode” in standards-compliant Web browsers to allow rendering of these noncompliant elements.

A successful open standard achieves and maintains the aim of having competing implementations of the same standard, making the substitution of alternative components possible in reality, not just theory. This essential state of affairs is much more likely where an open source implementation exists, for the following reasons [20,21]: (1) an open source implementation acts as a reference implementation, revealing standard specifications that are unnecessarily hard to implement or contain specification flaws, and (2) OSS tends to enjoy wide diffusion and dissemination, facilitating adoption of the standard.

Having an open source implementation of a standard therefore means both that the standard is more likely to be of high quality and that the standard is much more likely to become widely adopted. In fact, it has been observed that all successful open standards have OSS implementations [20]. Therefore, when creating or choosing a sustainable open standard it is very unwise to create or choose a standard without at least one open source implementation.

### Contemporary Health Care Information Systems Procurement Strategies and Standards

In the United Kingdom, the government chose to procure HIS centrally and implement them locally via five separate local service providers, who in turn were able to choose and change subcontractors [5]. The software being developed for use under NPfIT is proprietary. The government created an output-based specification [10], which was then tendered to interested contractors who employ programmers to write software that meet the specifications. Unfortunately, compared with OSS, this development model is often more expensive, less responsive to users, less secure, and more vulnerable to lock-in. In lock-in, a software purchaser loses the ability to switch software products because of the use of proprietary data formats or restrictive licensing conditions [22-24].

The United States has already developed an excellent HIS, the VA VistA hospital system, which directly serves or forms a core part of the software serving almost 30 million Americans [25]. Unfortunately, outside of the VA network of hospitals, uptake of HIS has been poor [7], the use of proprietary software is commonplace, and there has been a paucity of high-quality, affordable, and interoperable HIS [26]. Adoption of a VistA-derived OSS HIS platform and reference implementation allows competition to be based on service and support, reducing licensing costs while also providing an inclusive environment where creativity, innovation, and flexibility are not stifled by platform barriers [16]. Perhaps unsurprisingly, some observers are already predicting OSS HIS adoption will soon become widespread [27].

There is a power asymmetry between vendors and purchasers of proprietary software comparable to that of vendors and purchasers of used cars, which is a so-called “lemon market.” In this comparison there are two main points. First, the typical purchaser of a used car is in a weak position because he or she lacks knowledge about the technical fitness of the product, is blind to everything but price, and has no way of identifying poor-quality used cars, the “lemons.” Second, ongoing maintenance costs depend on the car’s design. If the car is designed in such a way that a specialist garage is required and generic replacement parts are hard to come by, the maintenance costs are high. A shrewd buyer may reduce this asymmetry by taking a warranty or having a mechanic look under the hood and inspect the car before buying. Such a buyer would also prefer more standard designs and generic parts, all else being equal, since these will tend to lower maintenance costs. Incidentally, there is one very important difference between the car market and the HIS market, which we will return to below. Namely, drivers are usually buyers in the car market but end users are not usually buyers in the HIS market.

In HIS procurement, purchasers are in a stronger position if they inspect, and allow others to inspect, the quality of the code; if they ensure that the programming code will be easily maintainable and that the data are stored in an established open format so that it will be cheap to get the data out and switch software when needed; and, finally, if they acquire the rights to the code, including the right to take it to another programmer or software company. In general, then, purchasers will be in a stronger position when they buy OSS rather than proprietary software.

There are a plethora of competing standards in HIS. Against this background DICOM stands out as a stunning success, and DICOM conformity is a standard part of just about every radiology product, software, or hardware. However, despite promising developments such as the US Nationwide Health Information Network [28], for most standards, open and closed alike, widespread conformity has not yet been achieved and this is to the detriment of interoperability.

### The Pros and Cons of Certification

Certification of standards in HIS has been mooted as essential to ensure interoperability and because of the safety-critical nature of HIS. The Certification Commission for Health Care Information Technology (CCHIT) is charged with certifying that American electronic health record systems meet standards in order that they qualify for Recovery and Reinvestment Stimulus Bill funds. Concern has been expressed that certification fees and other aspects of the process of certification, such as handling of versioning and a preference for comprehensive rather than modular systems, is a barrier to entry for OSS [28]. There has also been some controversy surrounding CCHIT’s relationship with vendors [29].

Certification of implementations of a standard is a choice. While it provides assurance to purchasers and users that a particular standard is met, the cost of certification must be borne and is often passed on to software developers. Despite being one of the oldest and most successful open standards bodies, the W3C does not have a certification process. In part this is because of the risk of alienating part of the industry or the Web community by adopting what could be seen as a policing or commercial role. It is also because of a concern that true vendor neutrality in certification is unachievable.

Certification may also restrict physicians in their own personal use of HIS. It has been observed that many physicians already use hand-held HIS and that psychological ownership is important for acceptance. Certification may undermine this [30,31].

It would be ironic if a healthy respect for the safety-critical nature of health care and the desire for interoperability leads to the proliferation of insufficiently open standards and to certification processes that close out OSS and stifle the development of effective HIS.

## Prerequisites of an Effective Health Care Information System

### General Prerequisites of Successful Information Systems

Three major reasons for IT project success across all sectors of the economy have been identified [32]: (1) extensive, informed, and continuing user involvement, (2) senior and executive manager support, (3) a clear and accurate requirements modeling strategy. Sadly, however, a common finding in software projects is that “significant budget and time-line overruns, under-delivery of value, and the outright termination of a project before completion are all forms of failure” [33,34].

Budget overspending and failure to deliver key features have plagued recent HIS projects, and cost remains a major issue for would be HIS purchasers [7,34].

### Prerequisites of an Effective Health Care Information System

In the first instance, we need a conservative or status quo HIS that mirrors, facilitates, and supports our current best practices. A system that demonstrably helps with the clinical workload in a reliable fashion is likely to have high spontaneous adoption rates. But as we would not wish our current clinical practices to be set in stone, so we should not wish our HIS to be static. Clinical acceptance is important and more likely to occur if significant process change is not required at the outset but instead is introduced after initial acceptance is secured, and in a stepwise fashion [9,25].

Returning to the major difference between car and HIS markets mentioned above, one might argue that better cars, from a driver perspective, result from driver choice. The driver is not compelled to buy a particular brand of car, and so car manufacturers have an incentive to make desirable cars – we leave unanswered what makes a desirable car. In the health care setting, choosing noncoercive implementation of an HIS could be an acid test of whether an HIS is of sufficiently high quality. Furthermore, employees may be permitted, and encouraged, to use rival but compatible HIS components, to promote desirable HIS. The assumption here is that health care professionals desire HIS that is usable, efficient, and helps to improve patient care.

We also need an affordable HIS software platform to be established to help coordinate and focus efforts on health transformational goals. The iPhone has been cited as a model successful platform [35] but a better model might be software, with Firefox or VistA as an example because these are less restrictive and more flexible platforms [36]. The implementation of an open source HIS platform will help to define and secure an open standard, as argued above [19,20]. This will make the addition and substitution of components possible, since modularity is an inherent feature of open source development. It will help to create a healthy market [35], as well as facilitating systems’ evolution, flexibility, and functional creativity [37].

Physician use of hand-held HIS should be encouraged as a means of making the end user the buyer and/or chooser of the HIS used, since this will tend to improve HIS. Therefore, smooth integration of hand-held HIS with hospital HIS should be a priority. Integration will be facilitated by an OSS HIS platform. Allowing individual physicians the freedom to choose the software that best suits them may help to drive meaningful use and innovative computer-aided practice [38].

## Why Open Source Software? Characteristics of Open Source Software and its Advantages

### General Argument

**Figure 2:**
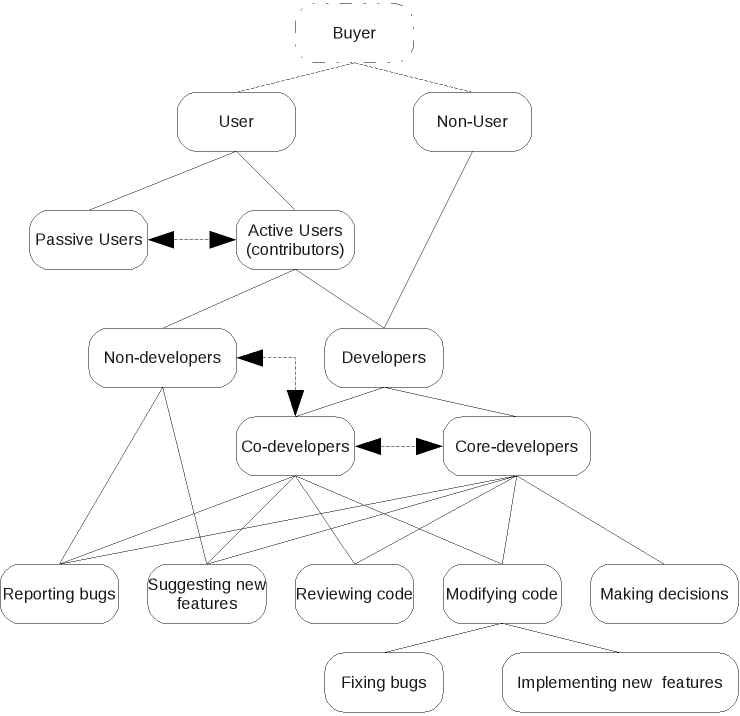
Open source software development process

The major single argument is that OSS empowers purchasers of software by making it easier for a given purchaser to change software products and/or software development teams, thus preventing lock-in and driving down costs [28]. However, important differences between the typical open source development model and proprietary development models provide a number of important additional arguments (Figures 2, Tables 1).

The arguments for OSS may be summarized as follows: (1) stronger position for purchaser, therefore lower costs, (2) software is superior (eg, usability, security, reliability) because of superior development model, quality of code can be checked, users can contribute, and contributors have many motivations (attracting highly motivated people to contribute “for free” is possible), and (3) facilitates open standards, encouraging competing implementations, strengthening the purchaser’s position, and leading to superior software.

In OSS development, there may or may not be a purchaser. A project may consist entirely of unpaid user-developers and users. If there are purchasers, they may employ core developers or a software company to write and release software to foster the formation of a community of user-developers. Existing specifications are usually adapted to meet user needs. Development benefits hugely from the involvement of the users.

This contrasts with proprietary development, in which there must be a purchaser. Either the software company creates a user specification for an imagined purchaser and then writes and markets the software to this purchaser, or it is paid to write software that meets a particular purchaser’s specification. Creating a comprehensive and accurate specification from scratch is costly. Users do not have access to the code so cannot contribute to it, and so any latent development skill possessed by users cannot be tapped. While a product continues to sell, the software company has little incentive to respond to individual user requests.

**Figure 3:**
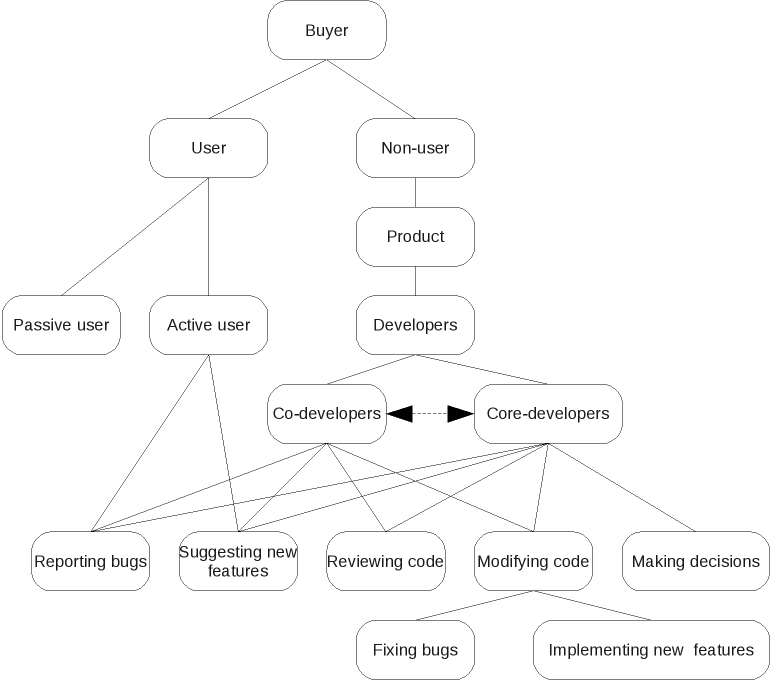
Proprietary software development process

**Table 1:** Comparison of proprietary and open source software development methods

Aspect	Proprietary software	Open source software
Software owner	Company, shareholders	Community, citizens
Foundations of product	Other products on market with a few distinct changes (analogy: me-too drug)	Existing tested code base (analogy: generic drug)
Pricing model	What the market will bear	Cost recovery
Development team	Professional programmers isolated from user base	Mix of professional and amateur programmers, often including users
Development team strategy	Cut and run, lock in to proprietary code	Code reuse, continuing quality improvement
Development team dynamics	Small, centralized, managed	Large, decentralized, meritocratic
Developer incentives	Salary, internal promotion	Community, recognition, contribution to application area ± salary & promotion
Method to test and assure quality	Internal synthetic test cases, team integrity	Real cases, user testing, open inspection by community
Driver to respond to user needs and requests	Market share	Community-prioritized need
Intellectual input	Small team, distorted by team dynamics	Wisdom of crowds

Successful OSS projects tap into the skills of the community that forms around them to suggest new features, report bugs, and modify the source code accordingly. A nascent developer community must have something testable to play with but, once formed, open source communities can put skilled time of much greater orders of magnitude into a problem [39]. The central argument to OSS development is that when everyone can inspect the source code, the software gets more scrutiny and more corrective feedback than a single development team can provide, leading to better software [40]. Reasons why this is so hinge on the characteristics of OSS and are multifactorial, but include [17,41] the following:

- Economic: a single proprietary software development team does not usually have the staff comparable to the size of the distributed communities involved in development of large OSS projects. OSS removes the need for duplication of programming effort (although it may occur anyway). Lock-in is prevented, leading to better long-term code security.
- Psychological: there is plurality of motivation, as members of an OSS community include individuals who may be more highly motivated [24] because they contribute for complex personal, rather than primarily financial, reasons such as peer recognition, as well as corporations motivated by financial gain.
- Social: OSS communities tend to be fluid, have a strong meritocratic culture, and foster creativity and innovation. OSS community meritocracies do break down in the usual human ways, but the licenses allow others to carry on through mechanisms such as “forking” (where a group of developers splits to form two groups and continue development along separate lines).
- Managerial: OSS projects tend to free community members from conventional managerial and bureaucratic constraints, facilitating innovation.
- Computer science/design related: Open source products have a high degree of modularity (necessitated by the distributed nature of development) and a high degree of interoperability.

**Table 2:** Implications of the differences in proprietary and open source software development methods

Aspect	Proprietary software	Open source software
Cost drivers	Competitors, value added	Development costs
Typical upgrade frequency	When competing products or serious bugs threaten – annual	When new release tested and robust – bimonthly
Use of proprietary tools, data formats	Frequent	Discouraged
Consequences of developer, company abandoning area	Catastrophic (even if source code deposited in escrow)	Not applicable
Software selling points	“Creeping featurism”	Robust, tested, user-centered software
Suitability for safety-critical applications	Only if relevant development and testing methods followed	Only if relevant user and developer community engaged
Risk of monopoly	Low to medium	Low
Ability of purchaser to influence quality, cost, upgrades	Low to medium	Medium to high
Training issues	Applications distinctive, specific training usually needed	Less training: generic look and feel so applications resemble one another
Process for tailoring to local needs	Pay remote software developer and wait	Ask local member of developer team and wait

## Barriers to the Adoption of Open Source Software

### General Barriers to Adoption of Health Care Information Systems

The major reported barrier to the adoption of HIS is cost [7]. Other barriers include physicians’ resistance to health care software because of the time cost of learning something new, fear of lawsuits, risk of data breaches, fear of automation and deprofessionalization, and poor track record of existing HIS [34,38,42].

### Particular Barriers to Adoption of an Open Source Software Health Care Information Systems

Lack of awareness and understanding of, and familiarity with, OSS is a major barrier to the adoption of OSS HIS [28], although this may be changing with the increasing recognition of the success of VistA and the adoption of VistA-derived OSS HIS platforms [27,36].

In many countries there is a lack of clear governmental support for OSS HIS, which may be attributable to a lack of awareness of the proven merits of OSS, the significant power wielded by lobby groups representing commercial or proprietary software developers and vendors, or a wish to protect tax revenue and employment generated by existing proprietary HIS markets. A number of myths have also circulated in the past about OSS such as it being more expensive, less secure, or riskier in terms of liability, which are debunked here and elsewhere [26].

It has been claimed that the total cost of ownership is often higher for OSS because of implementation costs. Lack of expertise and business drawbacks, including training investments and finding the right staff or the right business to outsource implementation and support, do have the potential to negatively influence total cost of ownership. However, many businesses in the service sector find that lower licensing costs and escape from vendor lock-in outweigh this [43-46].

It has been argued that OSS is inherently less secure than closed proprietary software. Arguments have included the claim that because the code is public in OSS an attacker can more easily find and exploit vulnerabilities. This is the “security through obscurity” argument, that systems that hide their inner workings from potential attackers are more secure. Security through obscurity alone completely fails when code is disclosed or otherwise discovered using tools such as debuggers or dissemblers [47].

Worse, it has been suggested that the cloak obscurity provides tends to encourage poor-quality code. Opening the source allows independent assessment of the security of a system, makes bug patching easier and more likely, and forces developers to spend more effort on the quality of their code [47].

The idea that using OSS is inherently riskier because one automatically become liable for any failings of the software is false. Typically a large organization will pay a contractor for an OSS implementation and support package. Many contractors providing OSS implementation and support offer legal indemnity to clients in exactly the same way as proprietary vendors [46].

### Particular Reasons to Adopt Open Source Software Health Care Information Systems

The general arguments for OSS previously summarized are that it (1) puts the purchaser in a stronger position, therefore lowering costs, (2) generates superior software (eg, usability, security, reliability) – because of the superior development model, quality of code can be checked, users can contribute, and contributors have multiple motivations (attracting highly motivated people to contribute “for free” is possible), and (3) facilitates open standards, encouraging competing implementations, strengthening the purchasers position, and leading to superior software.

These arguments obtain particularly for OSS HIS. In summary this is because (1) large sums of money are spent on HIS, which makes it easier for the purchaser to lever the advantages of OSS HIS, (2) OSS HIS development can benefit hugely from an existing large, talented, and highly motivated user base, and (3) existing proprietary HIS have not delivered as claimed, and an absence of OSS reference implementations has led to an absence of successful open standards, and in turn an absence of competing implementations.

Health care systems have large, highly trained technical work forces (there were 633,000 employed surgeons and physicians alone in 2006 in the United States [48], and there are approximately 1.3 million full-time workers in the UK NHS). Within these workforces are large numbers of individuals who will report software bugs and request new features in an environment where developers are responsive to this [16,28]. Even though a smaller number of individuals, perhaps one in a thousand, will be motivated and able to fix such bugs and implement new features, this still amounts to a critical mass of several hundred physicians and surgeons. These individuals are immersed in the nuances and intricacies of clinical practice and much better placed than external developers to make software that complements their work [16,23,28,49].

A team of paid core developers could ensure key features are delivered in a timely fashion, building on existing [40] open source medical software projects and preventing duplication of effort. Indeed, Ubuntu Linux, a highly successful open source operating system with over 6 million users, already follows a similar model.

The long-term security of the code base could be protected with a licensing arrangement that specifies that the code remain open and without restriction, allowing the government to readily employ a different team of programmers or businesses to continue development of the code should the need arise.

The complex personal motivation and values within OSS communities, such as healthy rivalry and respect for demonstrated excellence, are a useful match with those found in the medical profession and academia. Together with the informatics talent already demonstrated within the medical profession [50], health care systems can provide fertile ground for the growth of an OSS community. Such a community will facilitate clinical engagement with software and foster creativity [49], innovation, the development of IT skills within a health care system, and an HIS that fits with the needs of clinical users and workflows [22].

A high degree of modularity, together with openness, will help ensure the dependability of the safety and security-critical systems within health systems. Indeed, OSS is already used in a number of safety and security-critical systems, such as German traffic light controllers and American spaceships [22,51-53].

Several mature and function-rich exemplar OSS HIS already exist, including VistA, an electronic health record programmed by Federal (US) employees working for the VA. Development began in the 1970s and in its present form VistA serves approximately 30 million Americans and now is a de facto standard for HIS. VistA has been hailed as “the aspirin of electronic health records” [26], and its success can be attributed to the decentralized distributed team development model initially used. This model has been seen as a precursor to OSS development. The public domain VistA code base already serves as the basis for a number of both commercial and noncommercial leading OSS HIS, such as WorldVista and ClearHealth [16,25,26,28,54,55].

Public and professional awareness of OSS successes is limited because differences in the commercial model mean that OSS software is less often marketed to the general public. However, industrial-grade OSS successes include the Apache Web server, which represents 50% of the world’s Web server market and is supported and distributed by a number of large corporations including IBM and Oracle [40]. In fact, without realizing it, millions of people use Linux everyday when they surf the Internet, use Google, or use a host of other systems with embedded Linux, ranging from washing machines to automated teller machines.

Internationally, governments and businesses are more keenly aware of the benefits of OSS and the threat it poses to the existing US-dominated proprietary software market. The American Center for Strategic and International Studies tracks governmental policies on the use of OSS, and its July 2008 report describes 275 open source policy initiatives to date, 135 of which are within Europe. EU Commission policy recommends that OSS should be promoted among public administrations in terms of efficiency, productivity, and quality of their services and provides funding for the use of OSS in e-government and e-business solutions [53].

In 2004 the UK government announced that it will consider OSS solutions alongside proprietary ones in IT procurement, will only use products with open standards, and will seek to avoid lock-in to proprietary IT products and services [56].

Since 2003 the official US Department of Defense policy is that OSS solutions should be given equal consideration alongside proprietary ones in IT procurement. The US Navy has gone further and in 2007 recognized OSS as key to operational effectiveness [57,58]. Both organizations value safety, interoperability, and cost effectiveness in their IT systems, as do health care providers.

**Figure 4:**
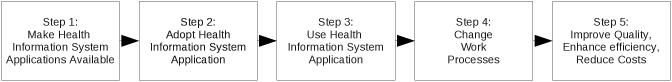
An illustration of the steps necessary before an HIS produces benefit [21]

The complexity of evaluating HIS and the lack of a good evidence base for the implementation of HIS have been noted. However, it has been recognized that for HIS to produce benefit it is first necessary that applications be available, adopted by institutions, and supported and used by clinicians (Figure 4) [21,59].

Rational HIS must aim to improve the quality of patient care, enhance efficiency, and reduce costs. This model emphasizes the importance of clinical engagement for the successful diffusion of HIS [9]. The rapid diffusion of OSS has been noted [24]and gives rise to the conclusion that OSS will benefit patients and professionals, and support the planned reforms of the health care system.

The particular suitability of OSS for HIS has already been mentioned briefly in the *British Medical Journal* [50] and, more recently, in an American Medical Informatics Association White Paper [25]. OSS continues to gain ground outside the health care setting, and in view of its manifest benefits, efforts to include it within the health care setting, and within HIS procurement strategies, must be renewed in order to maximize return on significant HIS investment.

In the future those who choose to invest in OSS HIS platforms, encourage individual physicians to use their own interoperable personal HIS, and take care not to create barriers to entry through regulation will be the first to fully realize the benefits of investment in HIS.

## Abbreviations

CCHIT: Certification Commission for Health Care Information Technology
DICOM: Digital Imaging and Communications in Medicine
HIS: health care information systems
IT: information technology
NHS: National Health Service
NPfIT: National Programme for Information Technology
OSS: open source software
VA: Veterans Administration
W3C: World Wide Web Consortium
